# Overexpression and Biological Function of Ubiquitin-Specific Protease 42 in Gastric Cancer

**DOI:** 10.1371/journal.pone.0152997

**Published:** 2016-03-31

**Authors:** Kun Hou, Zhenya Zhu, Yong Wang, Chunhui Zhang, Shiyong Yu, Qi Zhu, Bo Yan

**Affiliations:** 1 Department of General Surgery, Shanghai Pudong District People’s Hospital, Shanghai 201299, China; 2 Department of General Surgery, Punan Hospital, Pudong New District, Shanghai 200125, China; Seoul National University, REPUBLIC OF KOREA

## Abstract

Ubiquitin-specific protease 42 (USP42) is a member of deubiquitinating enzymes (DUBs). The alterations of DUBs are implicated in the pathogenesis of a wide variety of tumors. However, there are few studies on the expression and biological function of USP42 in gastric cancer (GC). Here, the expression levels of USP42 were significantly higher in GC tissues than in non-tumorous tissues. USP42 expression was significantly correlated with tumor size, TNM stage, lymph node metastasis and overall survival of patients with GC. Moreover, USP42 silencing in two GC cell lines, AGS and MKN-45, notably inhibited cell proliferation, but stimulated G1 phase arrest. The proteins promoting cell cycle progression (Cyclin D1, Cyclin E1 and PCNA) were down-regulated in USP42-suppressed cells. Moreover, inhibition of USP42 in GC cells impaired cell invasion via affecting the expression of matrix metalloproteinases (MMPs) and epithelial-mesenchymal transition (EMT) regulators. In conclusion, USP42 overexpression could be a potential prognostic marker for GC, regulate the survival and invasive properties of GC, and may represent a novel therapeutic molecular target for this tumor.

## Introduction

Gastric cancer (GC) is the fifth most common cancer[1] and the third leading cause of cancer death[2]. Currently known major risk factors for GC include Helicobacter pylori (H. pylori) infection, living environment, diet, genetic and immune factors, and chronic stomach diseases [3]. The prognosis among patients with GC is generally poor, because the tumor has often metastasized and most patients are elderly (median age is over 70 years) at the time it is diagnosed. The 5-year survival rate for GC is reported to be less than 25% [4]. It is of great clinical importance to identify sensitive diagnostic and prognostic markers of GC, investigate molecular mechanisms of GC development, and explore new therapy targets of this disease.

Ubiquitin-specific protease 42 (USP42) is a deubiquitinating enzyme (DUB) that is widely expressed in various human tissues [5]. Ubiquitination, a reversible post-translational modification, is involved in multiple cellular processes, such as cell cycle, DNA repair and apoptosis [6, 7]. Increasing evidence has demonstrated that altered DUB function is implicated in the pathogenesis of a wide variety of tumors [8]. Overexpression of USP9X, USP9Y, USP10 and USP25 was revealed in breast cancer by two-dimensional polyacrylamide gel electrophoresis and proteomics analysis [9]. A few studies have demonstrated that USP22 overexpression promoted cancer progression and poor prognosis of glioma, pancreatic cancer, cervical cancer and lung cancer [10–13]. USP42 has previously been found to be rearranged in acute myeloid leukemia [14]. However, to our knowledge, no investigation has been performed on the expression pattern and biological functions of USP42 in GC.

In the present study, USP42 mRNA levels in GC tissues were found to be remarkably higher to levels in controls. Further clinical characteristics analysis showed that expression level of USP42 was associated with overall survival of GC patients. We then applied RNA interference (RNAi) technology to knock down the expression of USP42 in two GC cell lines (AGS and MKN-45 cells), and investigated the proliferation, cell cycle and invasive capacity in both cell lines. Our data suggest that USP42 is a potent oncogene in GC, providing us with a future target for GC therapy.

## Materials and Methods

### Tissue samples

A total of 90 GC patients undergoing surgery at the Department of General Surgery, People’s Hospital, Pudong New District (Shanghai, China) between February 2007 and June 2009 were enrolled in this study. The median age of patients was 56 years (range: 34–68 years). All patients were given written informed consent. The study was approved by the independent ethics committee of Shanghai Pudong District People’s Hospital (Shanghai, China). Tumor tissue samples were obtained from all GC patients. Meanwhile, 42 matched non-tumorous samples located >3 cm away from the tumor were collected. All surgical samples were frozen in liquid nitrogen immediately after surgical resection, and stored at −80°C until RNA extraction.

### Cell lines

The cell lines derived from human gastric cancer, including AGS, SGC-7901, BGC-823, MKN-28 and MKN-45 were obtained from the Institute of Biochemistry and Cell Biology, Chinese Academy of Sciences (Shanghai, China). All cell lines were maintained in RPMI 1640 medium (Gibco, Grand Island, NY, USA) supplemented with 10% fetal bovine serum (FBS) and antibiotics at 37°C in a humidified atmosphere with 5% CO_2_.

### Silencing of USP42 by small interfering RNA (siRNA)

siRNA specific for human USP42 (5’-AUGGCCUCUGGUAUCAAAU-3’) were selected. A non-specific scramble siRNA sequence (siNC) was used as negative control. The siRNAs were transiently transfected into AGS or MKN-45 cells using lipofactamine 2000 (Invitrogen, Carlsbad, CA, USA) according to the manufacture’s instruction. Assays were performed 48 h after transfection.

### Real-time PCR

Total RNA was extracted using TRIzol Reagent (Invitrogen) according to the manufacturer’s instructions. Reverse transcription reaction was performed with random hexamer primers and a SuperScript Reverse transcriptase kit (Invitrogen). The resulting cDNA was used as template for real-time PCR performed with a standard SYBR Green PCR kit (Fisher Scientific, Rockford, IL, USA) on ABI7300 (Applied Biosystem, Foster City, CA, USA) thermal cycler. GAPDH was used as control of the input RNA level. All reactions were conducted using the following cycling parameters, 95°C for 10 min, followed by 40 cycles of 95°C for 15 s, 60°C for 45 s. To verify specific product amplification, the products were then subjected to dissociation curve analysis. The gene expression was calculated using the Δ Ct method. All data represent the average of three replicates. The sequences of specific primers were as follows: USP42 mRNA forward, 5’- ATGGAAAGCAGGGATGAC-3’, and USP42 mRNA-reverse, 5’- ACGCAGATTGGAACAGAG-3’; GAPDH mRNA forward, 5’- CACCCACTCCTCCACCTTTG-3’, and GAPDH mRNA reverse, 5’- CCACCACCCTGTTGCTGTAG-3’.

### Antibodies and Western blotting

Antibodies against CyclinD1, E-cadherin, β-catenin, Snail1 and GAPDH were purchased from Cell Signaling Technology (Danvers, MA, USA). Antibodies against USP42, CyclinE1, PCNA, and MMP-9 were from Abcam (Cambridge, MA, USA). Anti-MMP-2 was from Epitomics (Burlingame, CA, USA). Horseradish peroxidase-conjugated secondary antibodies were from Beyotime Biotechnology (Shanghai, China).

Cells were washed three times with PBS and then lysed in pre-cooled radioimmunoprecipitation (RIPA) assay buffer on ice for 10 min. After removal of cell debris by centrifugation (12,000g, 10 min), protein concentration of supernatants was measured by BCA protein assay kit (Thermo Fisher Scientific). After boiling for 5 min in sample buffer, equal amount of proteins of different groups were separated by SDS–PAGE, and transferred onto to a nitrocellulose membrane (Millipore, Bredford, USA). After blocking with 5% skim milk, the membranes were incubated with the primary antibodies at 4°C overnight with agitation, followed by incubation with corresponding secondary antibodies for 1 h at room temperature with agitation. Reactive protein was then detected using ECL chemiluminescence system (Bio-Rad, Richmond, CA, USA).

### Cell proliferation assay by CCK-8

The CCK-8 assay was performed by standard methods in 96-well dishes. Briefly, 3 × 10^3^ cells were seeded per well. At indicated time point, CCK-8 solution (10 μl in 100μl RPMI-1640 medium) was added to each well and incubated for 1 h. Absorbance at 450 nm was detected using a microplate reader.

### In vivo tumor bearing nude mice model

Animal experiments were approved and performed according to the guidelines of Animal Care and Use Committee of Shanghai Pudong District People’s Hospital (Shanghai, China). Twelve BALB/c nude mice aged 4–5 weeks old (SLAC Animal, Shanghai, China) were maintained under specific pathogen-free conditions using a laminar air-flow rack and had continuous free access to sterilized food and autoclaved water. Experiments were started after 1 week of acclimatization. AGS cells (2 × 10^6^) were subcutaneously injected into the right flank of nude mice to establish xenograft tumor-bearing model. Ten days after subcutaneous injection, mice were randomly divided into two groups (n = 6/group) and IV injected with USP42 siRNA or siNC containing formulations twice a week. The shortest and longest diameter of the tumor were measured with calipers at 4-day intervals, and tumor volume (mm^3^) was calculated using the following standard formula: (the shortest diameter)^2^ × (the longest diameter) × 0.5. 36 days after tumor placement, the mice were sacrificed by cervical dislocation and the tumors were recovered. The wet weights of each tumor were examined. During the experimental procedure, all mice were monitored every day. No mice died prior to the experimental endpoint.

### Cell cycle analysis

Cells were trypsinized, washed twice in PBS and fixed overnight at 4°C in ice-cold 70% ethanol. The cells were then washed twice in PBS, and incubated in propidium iodide (PI) staining buffer (5 μg/mL PI and 0.25 mg/mL RNase, Sigma, St. Louis, MO, USA) at room temperature for 30 min. Cells were then analyzed using a using a FACScan flow cytometry (BD Biosciences, San Jose, CA, USA). The percentages of cells in G0/G1, S and G2/M phase were determined by PI staining.

### Cell invasion assays

To measure the cell invasive potential of cells, Transwell assays were done using a Matrigel-coated Boyden chamber (BD Biosciences). Cells were serum-starved overnight, harvested, and resuspended in serum-free medium. Cells (1 × 10^5^) were then was added to the upper chamber. Medium containing 10% FBS was added to the lower chamber. After the cells were incubated at 37°C for 24 hours, the cells on the upper surface of the membrane were completely removed using cotton tips. The migrant cells attached to the lower surface were fixed in 4% paraformaldehyde and stained with 0.5% crystal violet. The number of migrated cells on the lower surface of the membrane was counted under a microscope in five fields at 100×.

### Bioinformatics analysis

Gastric cancer datasets were downloaded from the NCBI Gene Expression Omnibus database (Access ID: GSE26253) and The Cancer Genome Atlas (TCGA). To further investigate the biological pathways involved in gastric cancer pathogenesis through USP42 pathway, Gene set enrichment analysis (GSEA) was performed using the publicly available software from the Broad Institute at MIT (http://www.broad.mit.edu/gsea/software/software_index.html) as previously described [15]. For each gene set, GSEA defines an enrichment score (ES), which reflects the correlation between the gene set and the sample.

### Statistical analysis

Kaplan-Meier survival analysis was performed using Medcalc (Mariakerke, Belgium). The Statistical Package for Social Sciences (SPSS) version 17.0 (SPSS, Inc., Chicago, IL) was used for the other statistical analysis. Results of experiments are expressed as mean ± SD. Student’s t test was used to compare values of test and control samples. Chi-square test was used to identify differences between categorical variables. Statistically significant differences were defined as having a *P*<0.05.

## Results

### Expression of USP42 in gastric cancer

**Fig 1:**
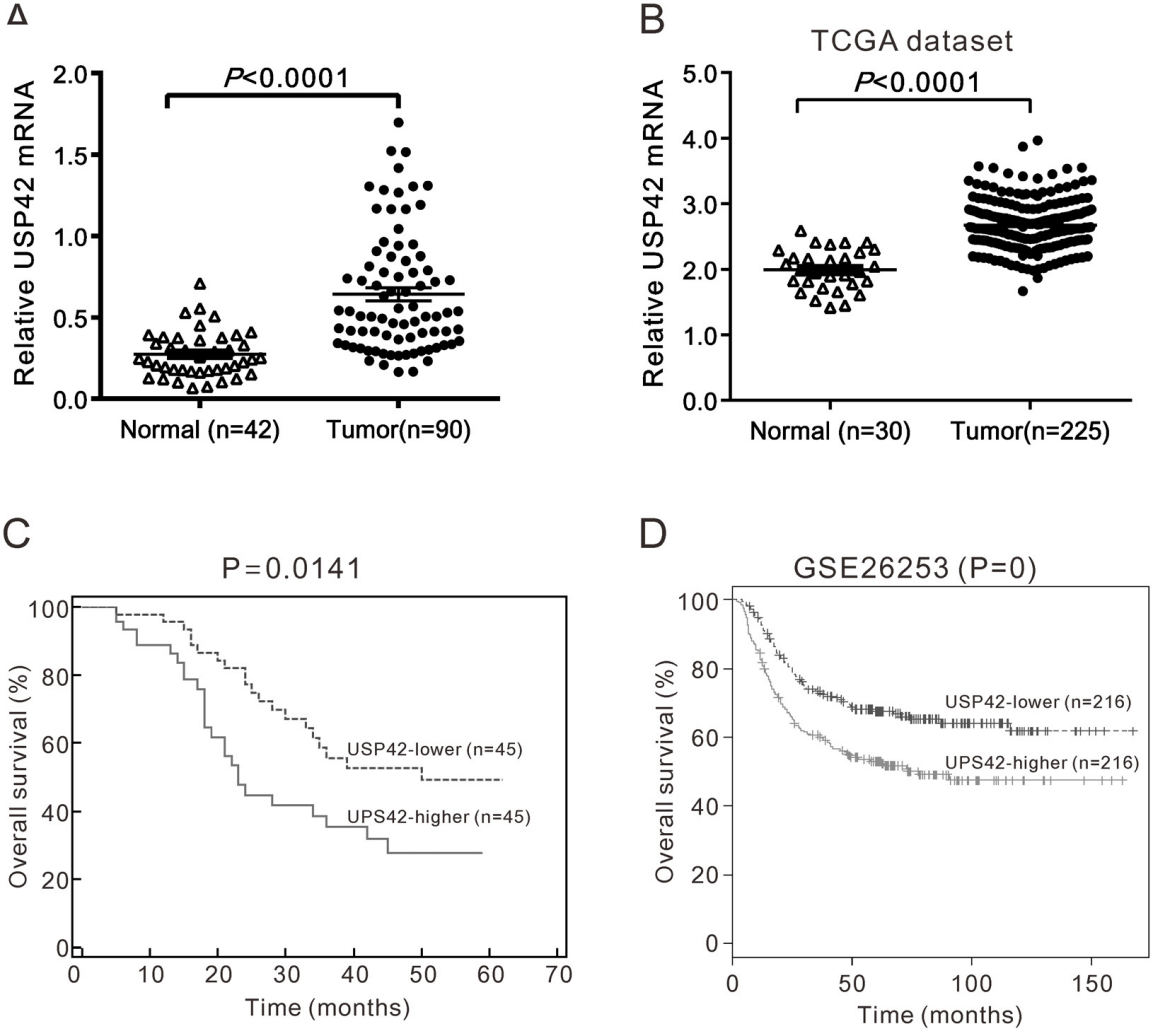
Increased USP42 expression correlated with poor survival of patients with GC. A. The mRNA levels of USP42 relative to GAPDH expression in GC and non-tumorous tissues were determined using real-time PCR (*P*<0.0001). B. The expression of USP42 in GC and normal tissues based on TCGA dataset (*P*<0.0001). C. Survival analysis showed that patients with USP42-higher expression tumors have a lower overall survival than those with USP42-lower expression tumors (*P* = 0.0141). D. Survival analysis on GSE26253 dataset.

To explore the expression of USP42 in GC, we performed real-time PCR analysis on GC (n = 90) and noncancerous tissue samples (n = 42). The relative expression of USP42 mRNA compared with GAPDH were calculated using the △Ct method. Clearly, USP42 mRNA expression was higher in GC tissues than in noncancerous tissues (Fig 1A). To further verify this finding, we re-analyzed microarray data from TCGA independent GC dataset. Fig 1B showed obvious overexpression of USP42 in human GC tissues as compared with normal tissues.

Using the mean value of 2^-ΔCt^ (0.550) as a cut-off between low-level and high level USP42 mRNA expression, 90 patients were classified into low (<0.550) and high USP42 expression subgroups (≥0.550). As shown in Table 1, USP42 was significantly associated with tumor size (*P* = 0.0328), TNM stage (*P* = 0.0059) and lymph node metastasis (*P* = 0.0184). However, there was no significant association between UP42 expression and other patient characteristics, including patients’ gender and age at diagnosis and tumor location. Kaplan-Meier survival analysis revealed that the overall survival time was significantly shorter in patients with higher USP42 expression than that in patients with lower USP42 expression (Fig 1C, P<0.05), which was further confirmed by survival analysis on ArrayExpress dataset (Access id: GSE26253, Fig 1D).

**Table 1:** Correlation of USP42 expression with patients’ features in gastric cancer.

Variables	All cases	USP42 mRNA		
		Low	High	*P* value
**Age at surgery**				
<55	26	11	15	0.4858
> = 55	64	34	30	
**Gender**				
Male	67	31	36	0.3339
Female	23	14	9	
**Tumor location**				
Upper half	32	17	15	0.8957
Lower half	42	20	22	
Whole	16	8	8	
**Tumor Size**				
> = 5 cm	51	31	20	0.0328*
< 5 cm	39	14	25	
**TNM**				
I+II	49	31	18	0.0059**
III+IV	41	14	27	
**Lymph node metastasis**				
Absent	52	32	20	0.0184*
Present	38	13	25	

*P<0.05,

***P<0.0001

### USP42 siRNA suppressed USP42 expression

**Fig 2:**
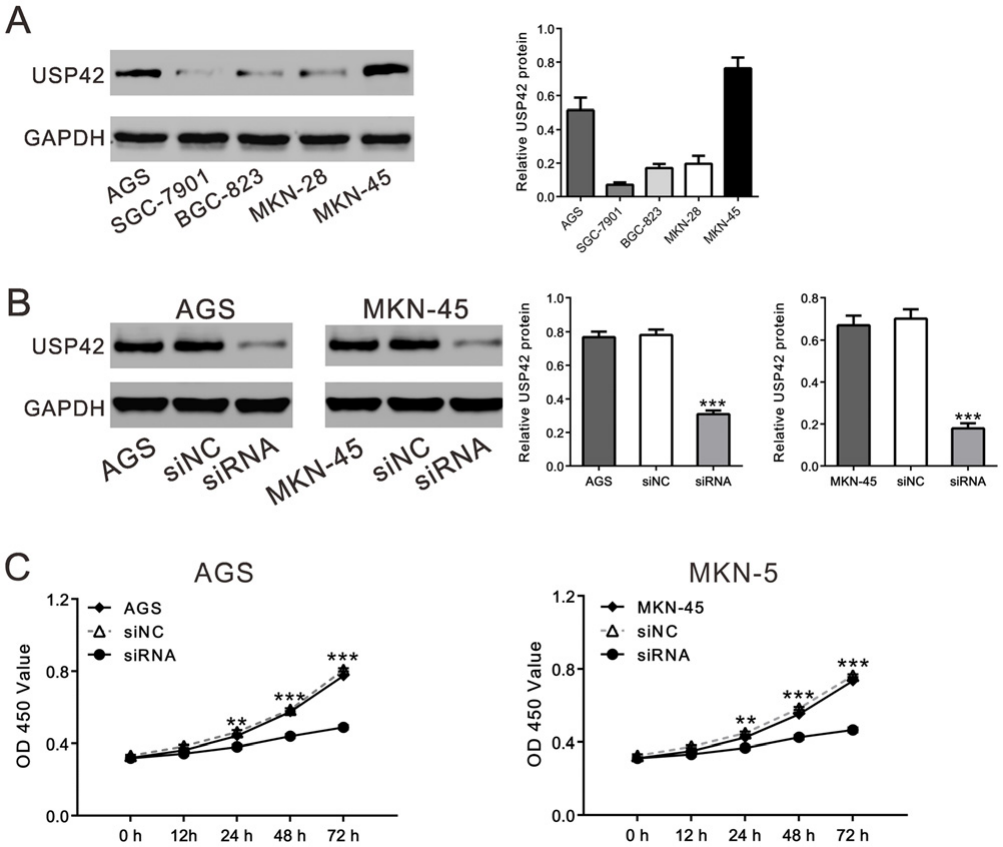
USP42 was important for GC cell viability. A. USP42 protein levels were determined in diverse GC cell lines by Western blot and normalized to GAPDH. B. USP42 silencing efficiency of siRNA transfection in AGS and MKN-45 cells was evaluated by Western blot. C. USP42 silencing by siRNA transfection resulted in growth inhibition as detected by CCK-8 assay in AGS and MKN-45 cells. Cells transfected with USP42 siRNA or non-silencing siRNA (siNC) for 48h, along with non-treated cells, were seeded in a 96-well plates, and cell viability was determined at indicated time points. * *P*<0.05, ** *P*<0.01, *** *P*<0.001 compared with siNC.

Comparison of various gastric cancer cell lines revealed that the expression level of USP42 protein in AGS and MKN-45 cells is higher than that of SGC-7901, BGC-823 and MKN-28 cells (Fig 2A). Therefore, AGS and MKN-45 cells were chosen for subsequent experiments. To explore the functions of USP42 on GC, we knock downed USP42 expression in GC cell lines by siRNA transfection. As shown in Fig 2B, siRNA specific for USP42 markedly suppressed USP42 expression in AGS and MKN-45 cells with a suppression ratio of 60.4% and 74.4%, respectively. We then analyzed whether suppression of USP42 expression would alter the growth rate of GC cells. As shown in Fig 2C, there was a significant decrease in growth rate of USP42-suppressed cells as compared with the siNC-transfected cells.

### USP42 siRNA suppressed tumor growth in nude mice

**Fig 3:**
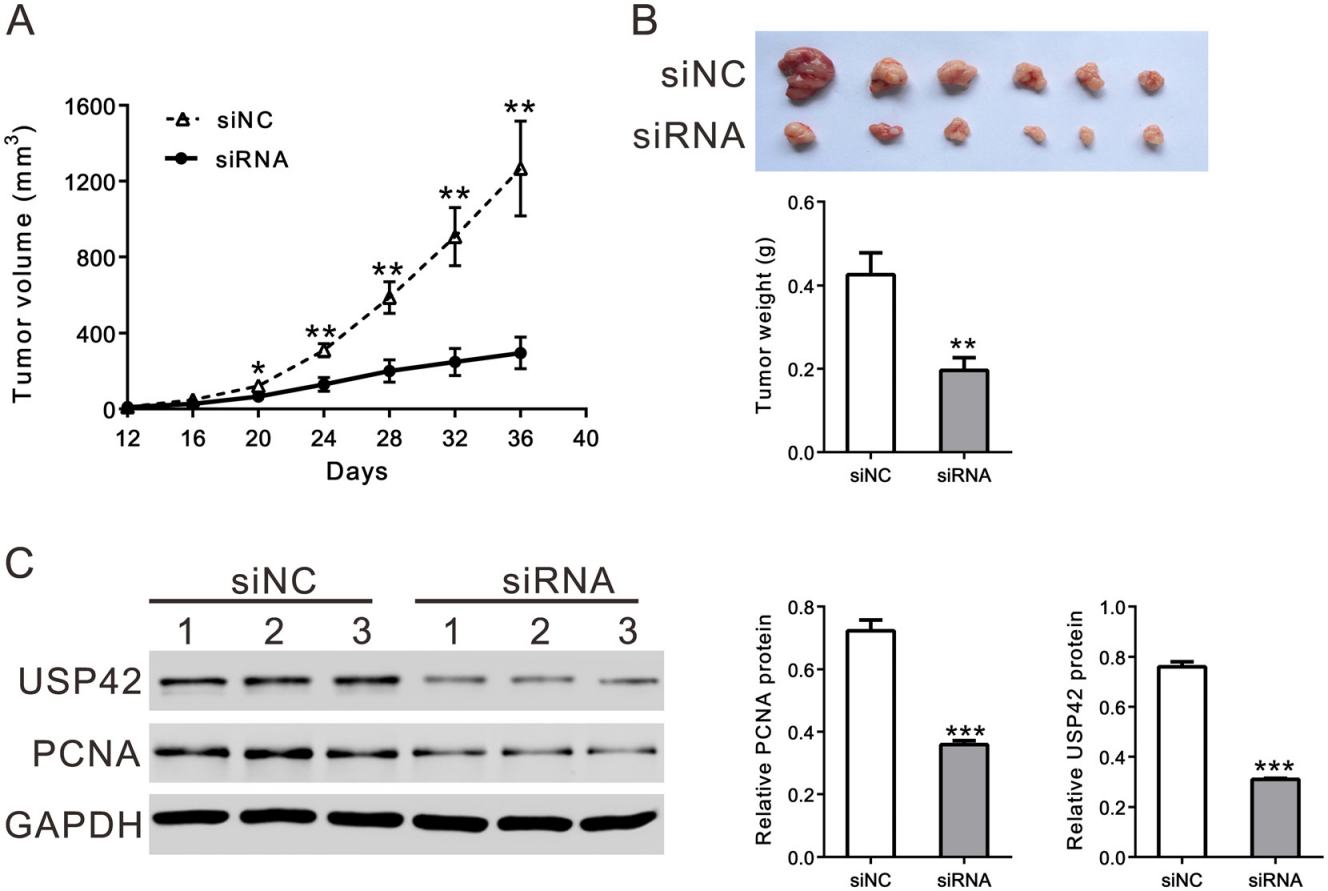
Knockdown of USP42 expression inhibited GC cell proliferation *in vivo*. A. Tumor volume was measured after USP42 siRNA (siRNA) or siRNA control (siNC) injection. B. Mice were sacrificed and tumors were recovered at 36 days after tumor placement. The pictures (upper panel) and weight (n = 6, expressed as mean ± SD, lower panel) of recovered tumors were shown. C. The expression of USP42 and PCNA in tumors from the nude mice was determined by western blot. * *P*<0.05, ** *P*<0.01, *** *P*<0.001 compared with siNC.

To determine the effect of USP42 siRNA on tumorigenicity in vivo, equal number of AGS cells was injected subcutaneously into nude mice and USP42-siRNA was IV injected after tumor formation. The tumor growth rate of mice injected with USP42-siRNA was significantly slower than that of mice injected with control siRNA (Fig 3A). The volume and weight of USP42-siRNA tumors was less than 25% that of control tumors (Fig 3B). Further, compared with control tumors, USP42 and PCNA were significantly decreased in tumors with USP42-siRNA injection (Fig 3C).

### Induced G0/G1 phase arrest in USP42-suppressed cancer cells

**Fig 4:**
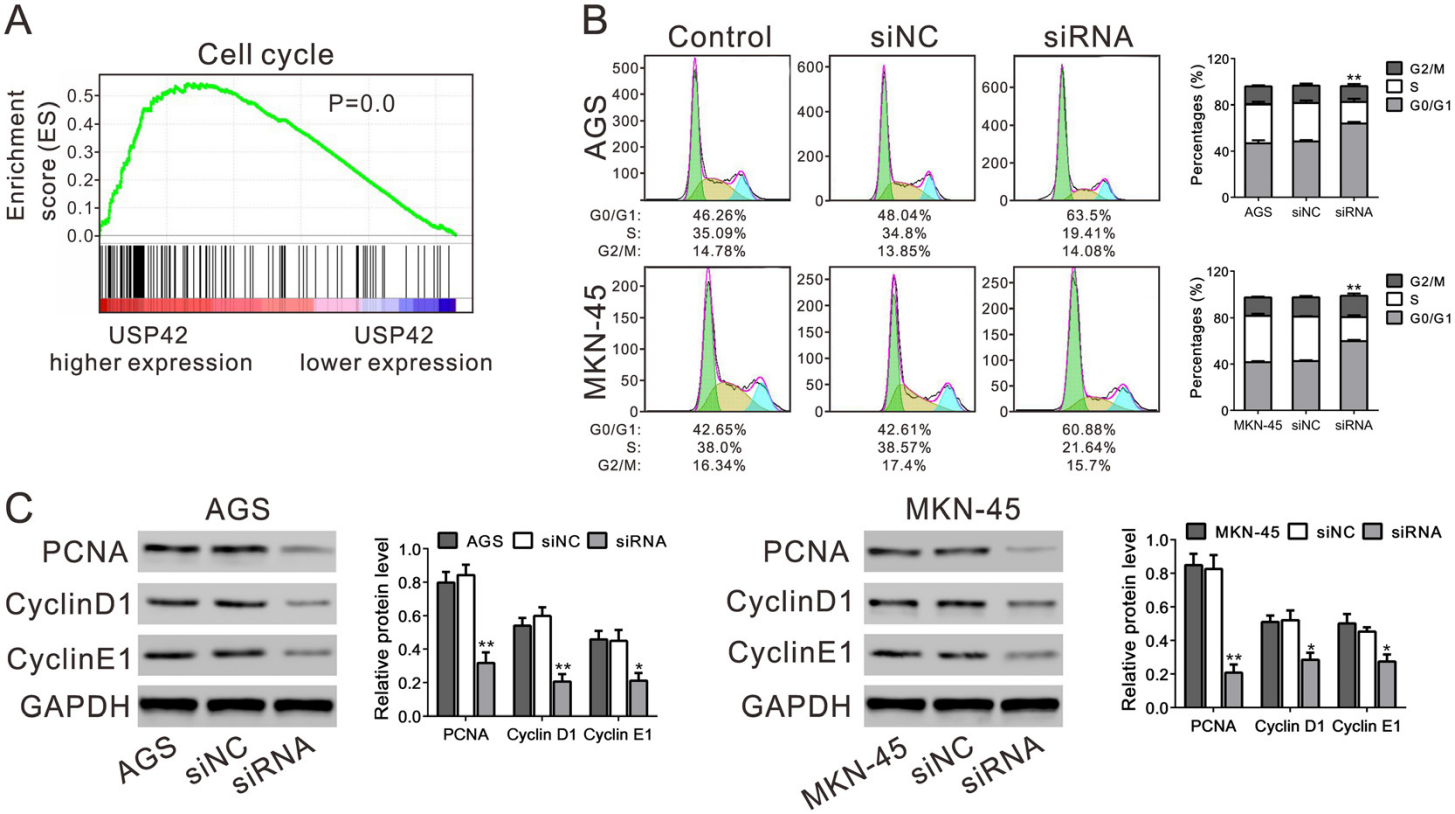
USP42 silencing induced G0/G1 phase arrest. A. GSEA analysis in GC patients with higher USP42 expression versus lower USP42 expression based on TCGA datasets. KEGG cell cycle pathway was associated with USDP42-higher expression. The enrichment score (ES, green line) reflects the correlation between cell cycle pathway and the sample. Black bars indicate previously known genes associated with cell cycle pathway. B. USP42 silencing induced a G0/G1 arrest in GC cells. FACS histograms and cell-cycle analysis of AGS and MKN-5 cells. Quantification of the percentage of cells in G0/G1, S, and G2/M phase was shown. C. Expression of key proteins in cell cycle was determined by western blot. All assays were performed in triplicate. * *P*<0.05, ** *P*<0.01, *** *P*<0.001 compared with siNC.

To find out how higher USP42 expression affected GC cell proliferation, we performed Gene Set Enrichment Analysis (GSEA) on TCGA dataset by analyzing the relation of USP42 expression and genes in Kyoto encyclopedia of genes and genomes (KEGG) cell cycle pathway. Interestingly, we found that higher USP42 expression was positively correlated with the KEGG cell cycle pathway in GC patients based on TCGA dataset (Fig 4A). We then analyzed cell cycle distribution of GC cells with USP42 knockdown. Suppressing USP42 expression reduced the S phase cell population and led to G1 arrest in AGS and MKN-45 cells (Fig 4B). To further explore the cellular mechanism underlying USP42-induced cell proliferation, the protein levels of cell cycle proteins were assessed. USP42 knockdown significantly reduced the expression of Cyclin D1, Cyclin E1 and PCNA (Fig 4C). Thus, these results revealed that a decrease in the level of USP42 expression inhibited Cyclin D1, Cyclin E1 and PCNA expression in GC cells, which may induce G0/G1 phase arrest, significantly reduce the number of S phase cells and inhibit cell proliferation.

### Low invasive potential of USP42-suppressed cancer cells

**Fig 5:**
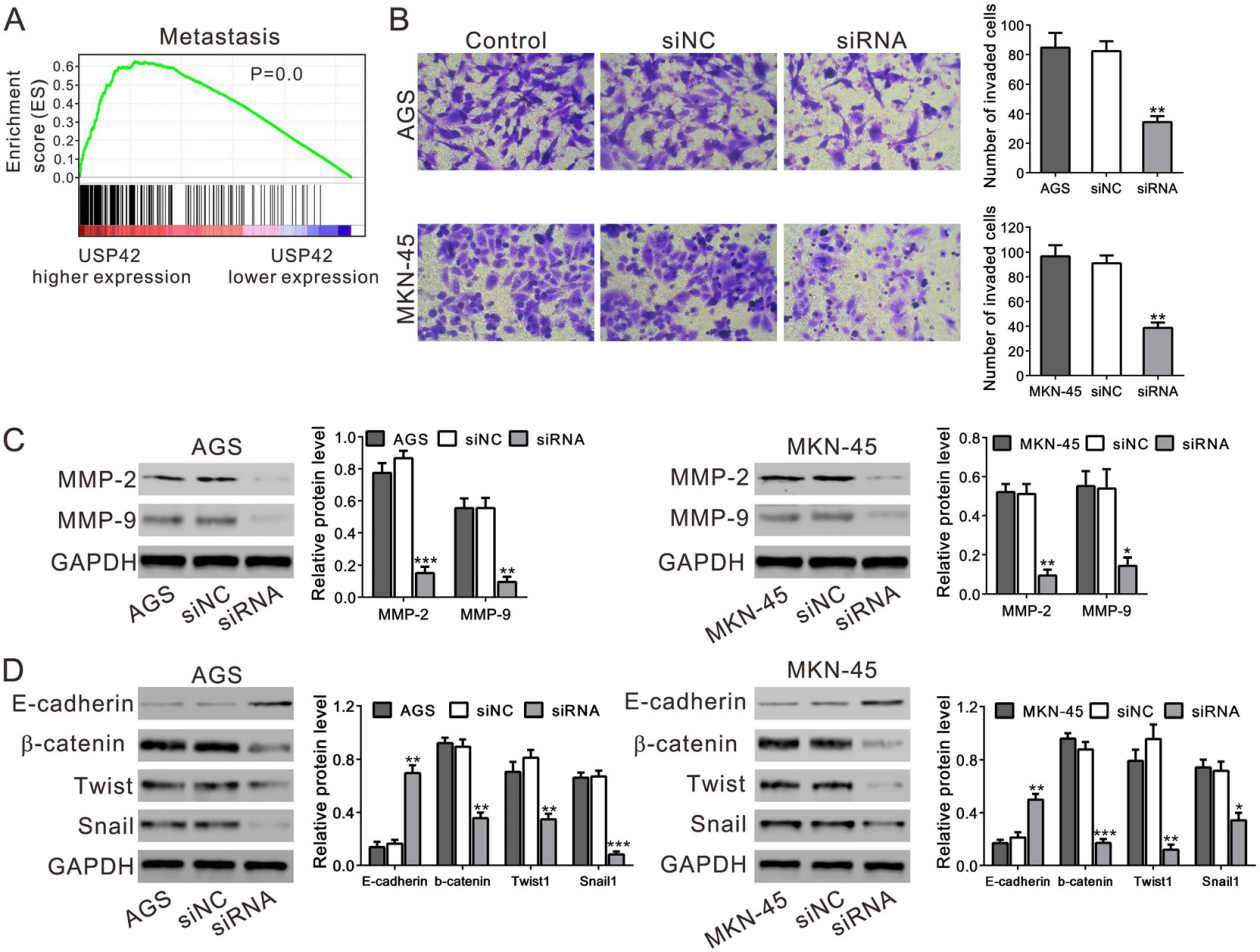
USP42 silencing inhibited cell invasion. A. GSEA analysis in GC patients with higher USP42 expression versus lower USP42 expression based on TCGA datasets. Cell metastasis pathway was associated with USDP42-higher expression. The enrichment score (ES, green line) reflects the correlation between metastasis pathway and the sample. Black bars indicate previously known genes associated with cell metastasis pathway. B. USP42 silencing decreased cell invasion as detected by in vitro invasion assay. C, D. Expression of key proteins in metastasis and EMT was determined by western blot. * *P*<0.05, ** *P*<0.01, *** *P*<0.001 compared with siNC.

GSEA also indicated that USP42 expression was positively correlated with metastasis (Fig 5A). To verify these findings in an in vitro assay, we examined the invasive potential of the USP42-suppressed cells using an in vitro Matrigel invasion assay (Fig 5B). The USP42-suppressed cells exhibited significantly less invasive potential than the siNC-transfected cells (*P*<0.01), suggesting that high expression of USP42 enhanced tumor invasiveness.

Degradation of the extracellular matrix through matrix metalloproteinases (MMPs), is a critical process during cell invasion [16]. As shown in Fig 5C, silencing of USP42 downregulated the expression of MMP-2 and MMP-9.

Further, the protein levels of epithelial-mesenchymal transition (EMT) pathway regulators, which are closely related to metastasis of tumor cells, were analyzed by Western blot (Fig 5D). Transfection of USP42 siRNA significantly reduced the expression levels of β-catenin, Twist and Snail1, while increasing E-cadherin.

## Discussion

Several members of the DUB family are known to contribute to carcinogenesis, including USP1 [17], USP2 [18], USP7 [19, 20] and USP22 [10–12, 21], while other DUBs are down-regulated in human cancers, including USP10 [22] and BAP1[23]. However, little is known about the expression pattern and biological functions of USP42, a DUB for p53[24] and histone H2B[25], in human cancers. In our study, we showed for the first time that GC tissues express high levels of USP42 mRNA compared with corresponding non-tumorous tissues. Moreover, USP42 expression was associated with tumor size, TNM stage, lymph node metastasis and overall survival of GC patients (Fig 1). Our findings provided valuable information for the clinical outcome prediction of patients with GC.

We presumed that USP42 may act as an oncogenic factor during GC development. We then applied RNAi technology, which is widely used in cancer research or cancer therapy, to knocking down USP42 expression in two GC cell lines (Fig 2B). Lowering USP42 expression effectively decreased cancer cell proliferation in vitro (Fig 2C) and *in vivo* (Fig 3). GSEA data revealed the association of cell cycle pathway with USP42 expression (Fig 4A). We then applied a cell-cycle analysis by FACS (Fig 4B) and expression analysis of Cyclin D1, Cyclin E and PCNA by western blot (Fig 4C). Our data revealed that USP42 siRNA had an inhibitory effect on GC cell growth via inducing G0/G1 arrest.

GC is one of the most common cancers and continued to be a serious public health problem in the world. High incidence of metastasis is still one of the main causes for the poor survival of GC patients [4]. GSEA on TCGA dataset revealed the association of metastasis pathway with USP42 expression in GC (Fig 5A). Further, in vitro invasion assay pointed out that USP42 knockdown decreased the invasive ability of immortalized GC cell lines (Fig 5B). Thus, our data indicated that elevated expression of USP42 in GC may promote tumor metastasis and is associated with the clinical outcome of GC patients. Next, we tried to explore the underlying mechanisms by evaluating the expression of MMPs and EMT regulators in USP42-suppressed cancer cells. MMP-2 [26] and MMP-9 [27] are known to mediate the degradation of the extracellular matrix and are related with lymph node metastasis of GC. EMT is involved in complex pathogenesis of tumors [28]. Here, silencing of USP42 induced the expression of the main factor of EMT (E-cadherin), but decreased the expression of MMPs and three known inducers of EMT (β-catenin, Twist and Snail1) (Fig 5C and 5D). These discoveries suggested the role of USP42 as an invasion promoter gene via affecting the expression of MMPs and EMT regulators, although further studies are required to clarify the exact mechanisms.

In summary, the present study demonstrated that USP42 expression was significantly increased in GC tissues. The increased USP42 expression might be important for tumor progression and metastasis of GC, and can serve as a prognostic marker for this disease. Our in vitro findings showed that silencing of USP42 inhibited cell proliferation via inducing G0/G1 arrest and suppressed cell invasion via MMPs and EMT regulators. Thus, USP42 may be a novel therapeutic molecular target for GC.
